# SCG5 and MITF may be novel markers of copper metabolism immunorelevance in Alzheimer’s disease

**DOI:** 10.1038/s41598-024-64599-z

**Published:** 2024-06-13

**Authors:** Xianbo Zhuang, Yitong Xia, Yingli Liu, Tingting Guo, Zhangyong Xia, Zheng Wang, Guifeng Zhang

**Affiliations:** 1 Department of Neurology, Liaocheng People’s Hospital and Liaocheng Hospital affiliated to Shandong First Medical University, Liaocheng, China; 2 School of Rehabilitation Medicine, Jining Medical University, Jining, China; 3 State Key Laboratory of Dampness Syndrome of Chinese Medicine, Shandong Sub-Centre, Liaocheng, China; 4 Department of Neurosurgery, Liaocheng Traditional Chinese Medicine Hospital, Liaocheng, China; 5 Department of Neurology, the Second People’s Hospital of Liaocheng, Liaocheng, China

**Keywords:** Computational biology and bioinformatics, Neuroscience, Biomarkers, Dementia

## Abstract

The slow-developing neurological disorder Alzheimer’s disease (AD) has no recognized etiology. A bioinformatics investigation verified copper metabolism indicators for AD development. GEO contributed AD-related datasets GSE1297 and GSE5281. Differential expression analysis and WGCNA confirmed biomarker candidate genes. Each immune cell type in AD and control samples was scored using single sample gene set enrichment analysis. Receiver Operating Characteristic (ROC) analysis, short Time-series Expression Miner (STEM) grouping, and expression analysis between control and AD samples discovered copper metabolism indicators that impacted AD progression. We test clinical samples and cellular function to ensure study correctness. Biomarker-targeting miRNAs and lncRNAs were predicted by starBase. Trust website anticipated biomarker-targeting transcription factors. In the end, Cytoscape constructed the TF/miRNA-mRNA and lncRNA-miRNA networks. The DGIdb database predicted biomarker-targeted drugs. We identified 57 differentially expressed copper metabolism-related genes (DE-CMRGs). Next, fourteen copper metabolism indicators impacting AD progression were identified: CCK, ATP6V1E1, SYT1, LDHA, PAM, HPRT1, SCG5, ATP6V1D, GOT1, NFKBIA, SPHK1, MITF, BRCA1, and CD38. A TF/miRNA-mRNA regulation network was then established with two miRNAs (hsa-miR-34a-5p and 34c-5p), six TFs (NFKB1, RELA, MYC, HIF1A, JUN, and SP1), and four biomarkers. The DGIdb database contained 171 drugs targeting ten copper metabolism-relevant biomarkers (BRCA1, MITF, NFKBIA, CD38, CCK2, HPRT1, SPHK1, LDHA, SCG5, and SYT1). Copper metabolism biomarkers CCK, ATP6V1E1, SYT1, LDHA, PAM, HPRT1, SCG5, ATP6V1D, GOT1, NFKBIA, SPHK1, MITF, BRCA1, and CD38 alter AD progression, laying the groundwork for disease pathophysiology and novel AD diagnostic and treatment.

## Introduction

Alzheimer’s disease (AD), a prevalent and highly debilitating form of dementia among the elderly population, constitutes approximately 50–70% of all cases of dementia in this age group. Its impact extends to the physical and mental well-being of approximately 40–50 million individuals globally^1,2^. The prevalence of AD is increasing due to the global phenomenon of population aging^3^. Projections indicate that the number of individuals affected by AD will triple by the year 2050^2^. This anticipated rise in AD cases poses significant challenges and burdens for both the global public health system and the socio-economic landscape.

The precise origin of AD has yet to be fully understood, and it primarily manifests as a degenerative condition affecting the central nervous system^4^. This disease is characterized by a gradual decline in cognitive function and behavioral abilities. The primary pathological characteristics of this condition encompass the accumulation of amyloid-β plaques and the atypical phosphorylation of Tau proteins, resulting in the formation of neurofibrillary tangles (NFT)^5^. Currently, there is no known cure for AD; however, a combination of therapeutic interventions exists to mitigate symptoms and decelerate the advancement of the illness. Hence, the identification of biomarkers associated with the progression of AD holds great importance in enhancing our comprehension of the disease’s molecular mechanisms and facilitating the development of novel therapeutic targets.

Copper, being the most prevalent alkaline excess metal essential for human physiological functions, plays a crucial role in numerous physiological processes such as antioxidant defense and neurotransmitter synthesis^6–8^. Hence, the investigation into the correlation between dysregulation of copper homeostasis and AD is increasingly gaining attention in academic research. Copper levels, both bound and free, are higher in the brains of AD patients compared to healthy controls^9,10^. Copper ions at low concentrations bind to tyrosine residues at the N-terminal end of the A peptide chain, causing oxidative modification that promotes plaque deposition and oxidative neurotoxicity^11^, thereby inducing A aggregation. Copper channels with synaptic proteins and neurotransmitter receptors form myelin sheaths and regulate synaptic activity^12,13^. The maintenance of normal copper metabolism is thus essential for the avoidance of AD pathology. Less is known about the role of genes involved in copper metabolism in the development of AD.

Hence, this study employed an integrated bioinformatics approach utilizing public database data to ascertain significant copper metabolism-related genes, referred to as biomarkers, in the advancement of AD. RT-PCR was conducted on peripheral blood samples obtained from patients diagnosed with AD in order to confirm the expression of specific genes. The experimental findings provided support for the initial bioinformatics screening conducted.

## Results

### Differentially expressed CMRGs (DE-CMRGs) associated with AD

**Figure 1:**
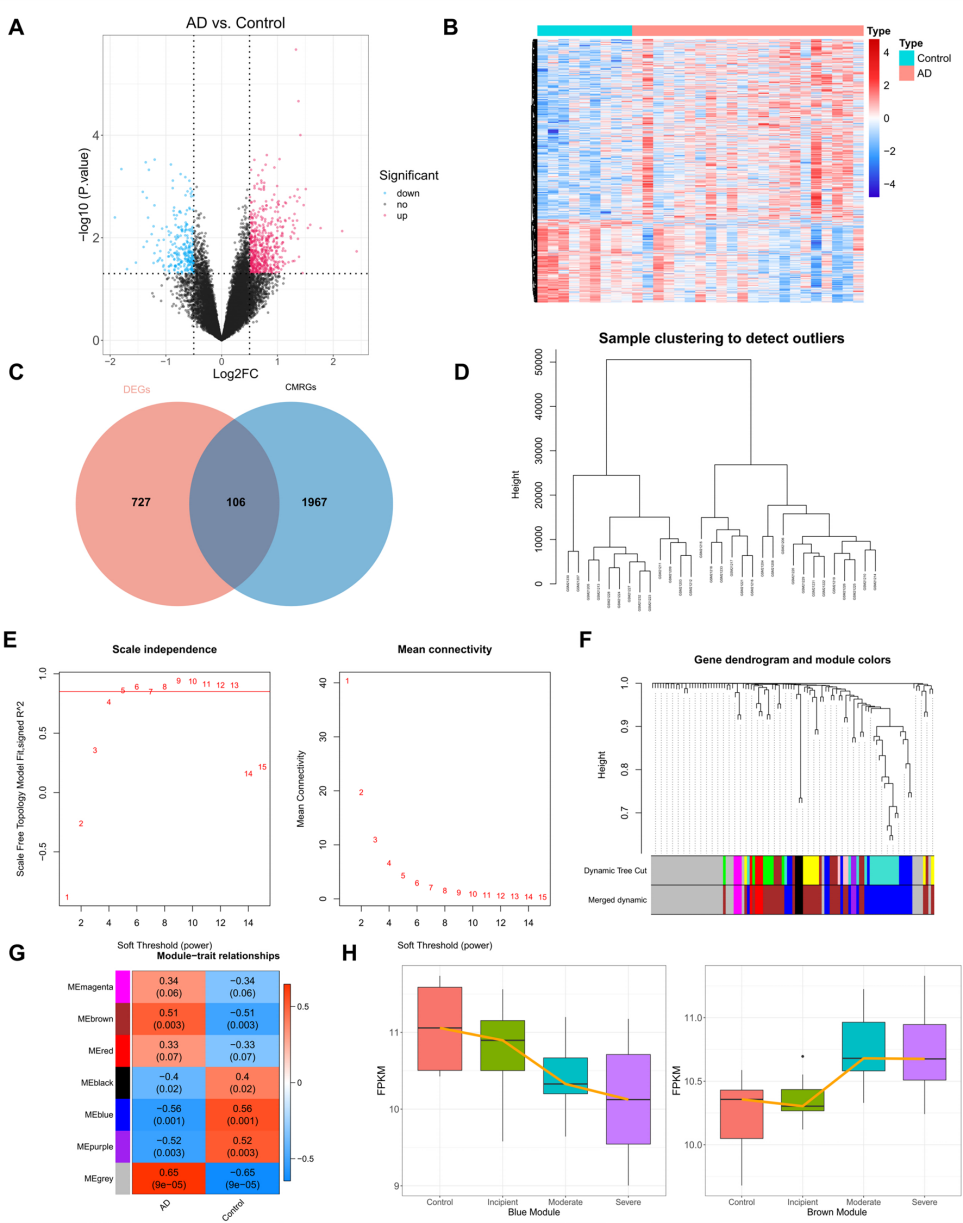
DE-CMRGs associated with AD (**A**) Volcano plot of differential genes, red indicates up-regulation in AD, blue indicates down-regulation. (**B**) Differential gene heat map, red indicates high expression, blue indicates low expression. (**C**) Intersection of copper metabolism genes with screened differential genes. (**D**) Sample clustering diagram, where each branch represents a sample and the vertical coordinate is the height of the hierarchical clustering. (**E**) Soft threshold analysis showed that gene associations were most consistent with the scale-free distribution when β = 10. (**F**) Clustering dendrogram of genes, dissimilarity based on the topological overlap, and assigned merged module colors. (**G**) Heat map of the relationship between gene modules and traits using AD and normal as phenotypes. The darker the color, the higher the correlation, red is a positive correlation, and blue is a negative correlation. (**H**) Expression of brown module and blue module genes in different clinical samples.

Using |log_2_FC|≥ 0.5 and adjusted p-value ≤ 0.05, we identified 833 DEGs (AD vs. control) from the GSE1297 dataset, with 571 highly expressed and 262 lowly expressed genes in AD (Fig. 1A-B, Supplementary Table 3). Differentially expressed genes (DEGs) were crossed with 2073 CMRGs to create 106 DE-CMRGs (Fig. 1C, Supplementary Table 4). Next, WGCNA added 106 DE-CMRGs. Figure 1D shows that sample clustering was unnecessary. Ten was the best soft threshold (R^2^ = 0.85) to maximize scale-free gene interactions (Fig. 1E). Next, dynamic tree cutting and module merging produced 7 modules (Fig. 1F). AD correlations were calculated for each module. The brown module had the highest positive correlation with AD (r = 0.51, p-value = 0.003), and the blue module had the highest negative correlation (r = − 0.56, p-value = 0.001) (Fig. 1G). Thus, 30 brown module genes and 27 blue module genes were essential module genes, or DE-CMRGs associated with AD (Supplementary Table 4). We calculated gene expression profiles in the brown and blue modules in AD samples (incipient, moderate, and severe). Figure 1H shows AD expression trends in box plots based on expression profiles. According to Weighted Gene Co-expression Network Analysis (WGCNA) (Fig. 1H), the blue module’s gene expression decreased and the brown module’s increased with AD.

### The function of DE-CMRGs associated with AD

**Figure 2:**
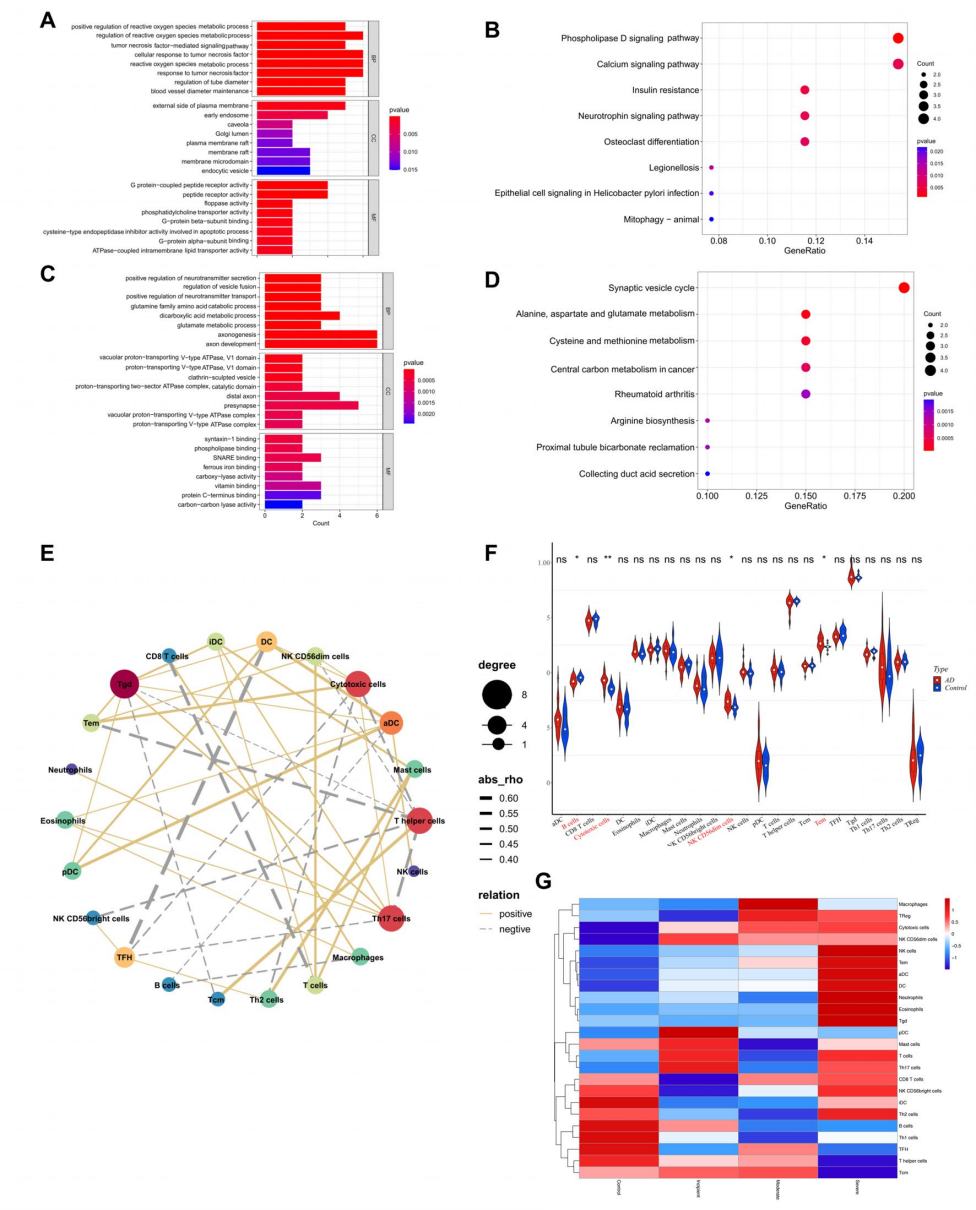
The function of DE-CMRGs associated with AD (**A**) GO enrichment analysis of brown module genes. (**B**) KEGG enrichment analysis of the brown module gene. (**C**) GO enrichment analysis of blue module genes. (**D**) KEGG enrichment analysis of the blue module gene. (**E**) The network displayed the correlation among 22 different types of immune cells. Bubble sizes represent scores, colors represent different cells. (**F**) Violin plot of the percentage of immune cells in the AD and normal groups. (**G**) Changes in immune cell infiltration during the AD disease process.

Functional enrichment analysis was executed to probe further the part of the DE-CMRGs related to AD. As displayed in Supplementary Table 5, 922 GO items (819 BP items, 28 CC items, and 75 MF items) and 20 KEGG pathways were derived based on 30 genes in the brown module. The top 8 items under each classification were shown in bar graphs (Fig. 2A-B). We observed that the genes above were mainly linked to reactive oxygen metabolism-related, immune-related biological processes, neuron apoptotic processes, phospholipase D signaling pathways, neurotrophin signaling pathways, calcium signaling pathways, and neuroactive ligand-receptor interaction. Meanwhile, 539 GO items (432 BP items, 46 CC items, and 61 MF items) and 23 KEGG pathways were enriched based on 27 genes in the blue module (Supplementary Table 6). The top 8 items under each classification were showcased in bar graphs (Fig. 2C-D). We noted that the genes above were mainly linked to neurotransmitter transport-related, axon development-related biological processes, synaptic vesicle cycle, HIF-1 signaling pathway, mTOR signaling pathway, and GABAergic synapse.

### The immune cells associated with AD

Since multiple immune-related biological processes were connected with DE-CMRGs related to AD, we next applied single sample gene set enrichment analysis (ssGSEA) to explore the immune cells that differ between AD and standard samples. The correlation between 22 types of immune cells was presented in the network (Fig. 2E). As shown in Fig. 2F, the scores of cytotoxic cells, NK CD56 cells, and Tem were elevated in AD, while the B cells were superior in the control. The score of each immune cell type in different grades of AD samples (incipient, moderate, and severe) was displayed in a bubble diagram (Fig. 2G).

### The biomarkers identified from DE-CMRGs associated with AD

**Figure 3:**
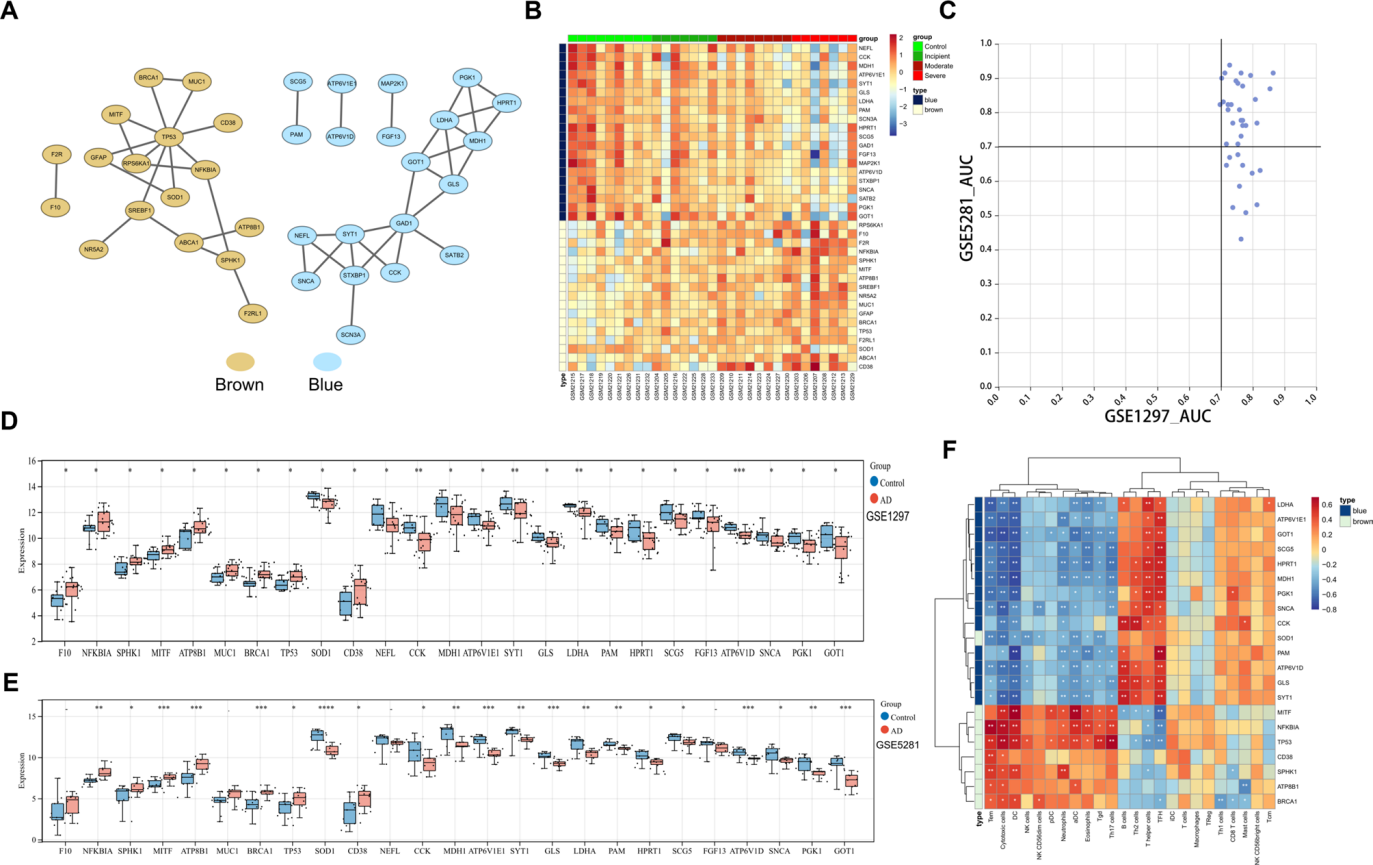
The biomarkers identified from DE-CMRGs associated with AD (**A**) PPI network for brown and blue modules. (**B**) A heatmap showed these 37 genes’ expression in control and early, moderate, and severe AD samples. (**C**) AUC values of ROC curves for these 37 genes in GSE1297 and GSE5281. (**D**) Differential expression of 25 genes in GSE1297 between AD and healthy controls. (**E**) Differential expression of 25 genes in GSE5281 between AD and healthy controls. (**F**) Heatmap of the correlation between 21 genes and immune cells.

To probe the linkage among the genes in the brown and blue modules, a PPI network containing 37 genes was generated (Fig. 3A). The expression of these 37 genes in control and different grades of AD samples (incipient, moderate, and severe) were shown in a heatmap (Fig. 3B). To identify genes with potential diagnostic value further, we calculated the Area Under the ROC curve (AUC) values of the receiver operating characteristic (ROC) curves for these 37 genes in the GSE1297 dataset and the external independent dataset GSE5281. As shown in Fig. 3C, 25 genes with AUC more significant than 0.7 in both datasets were identified. Among the 25 genes, the expression of F10, MUC1, NEFL, and FGF13 was not significantly different between AD and control samples in the GSE5281 dataset (Fig. 3D-E). Hence, we calculated the correlation between the remaining 21 genes and immune cells. As illustrated in Fig. 3F, NFKBIA was highly correlated with Tem, CCK was positively associated with B cells, and TP53 was positively associated with Cytotoxic cells.

**Figure 4:**
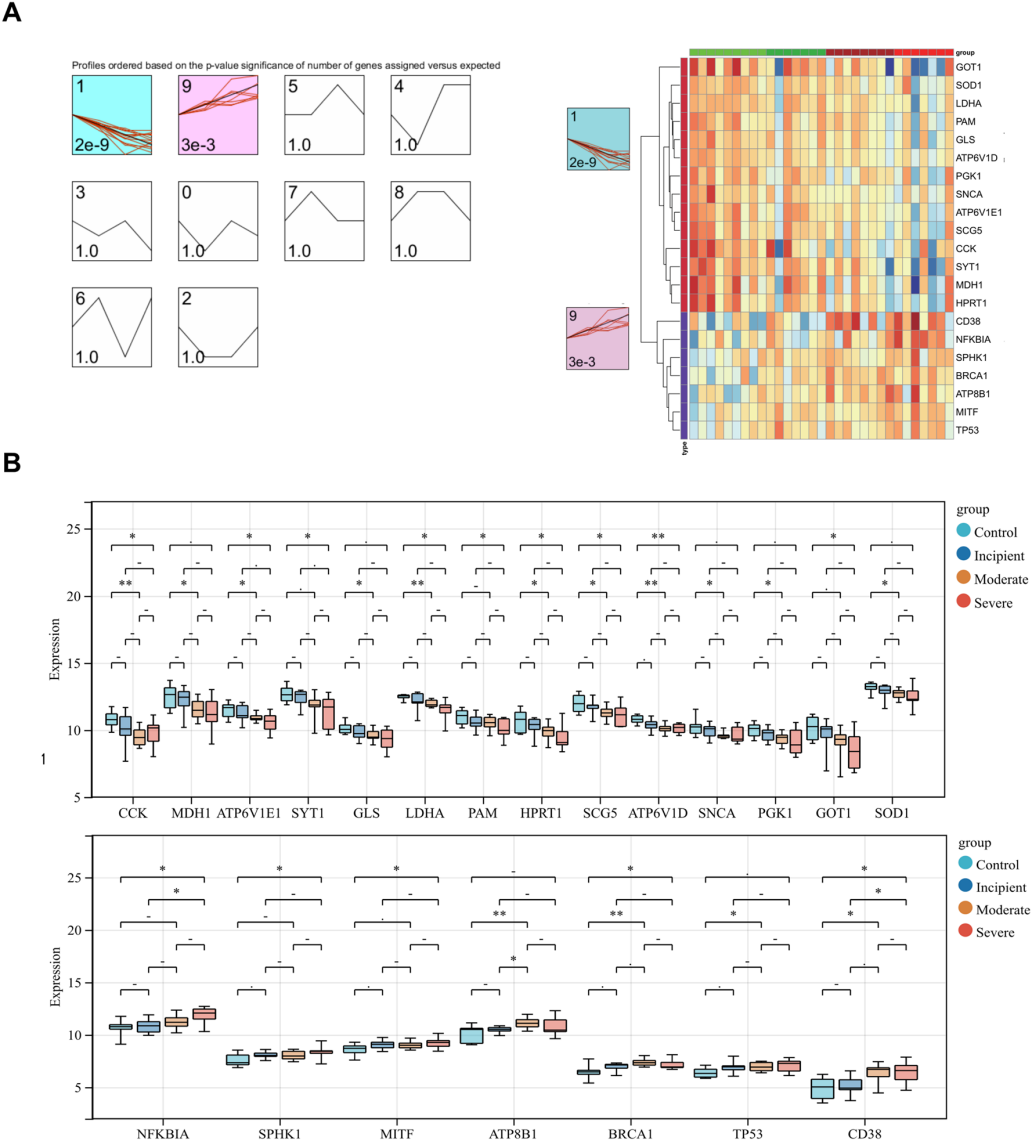
STEM to cluster the above 21 genes (**A**) STEM temporal clustering results, where the black line represents the overall trend, the red line represents individual genes, the upper left corner represents the number of clusters, the lower left corner represents the P-value, and the colored clusters represent significantly clustered genes. (**B**) The STEM clusters’ expression of important genes that differed significantly between controls and severe AD patients yielded 14 final genes.

**Figure 5:**
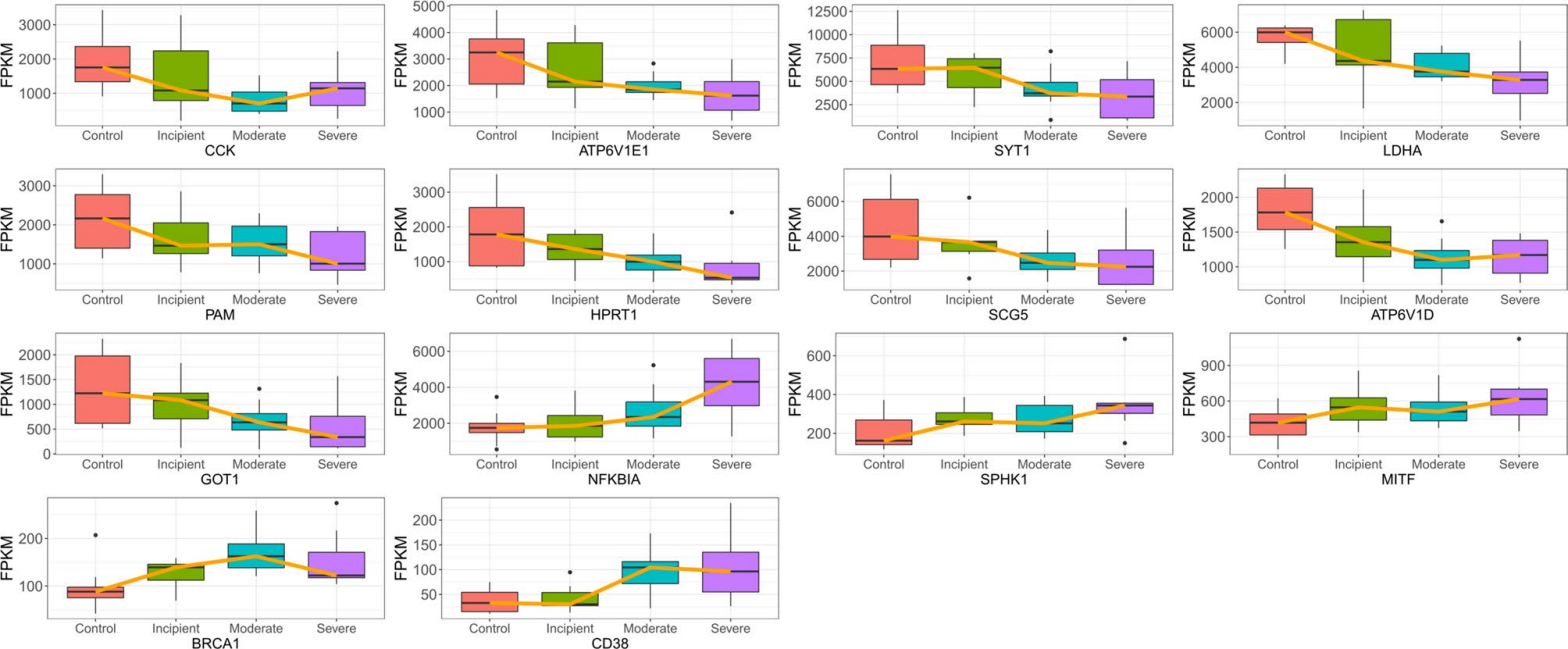
Expression of 14 genes in different disease stages of AD With increasing AD degree, CCK, ATP6V1E1, SYT1, LDHA, PAM, HPRT1, SCG5, ATP6V1D, and GOT1 decreased and NFKBIA, SPHK1, MITF, BRCA1, and CD38 increased.

Next, we utilized STEM to cluster the above 21 genes to explore their changes during disease progression. As exhibited in Fig. 4A, the 21 genes were clustered into two clusters, with 14 genes in cluster 1 tending to decrease as the disease progressed and seven genes in cluster 9 tending to increase as the disease progressed. The expression of the genes in these two clusters in control and different grades of AD samples was presented in the heatmap and box plots (Fig. 4B). We found significant differences in the expression of 14 genes between severe AD samples and control samples in which the expression of CCK, ATP6V1E1, SYT1, LDHA, PAM, HPRT1, SCG5, ATP6V1D, and GOT1 gradually decreased with increasing AD degree, and the presentation of NFKBIA, SPHK1, MITF, BRCA1, and CD38 gradually increased with increasing AD degree (Fig. 4B, Fig. 5). We defined these 14 genes as biomarkers associated with copper metabolism that impacted AD progression.

### Results of in vivo validation

**Figure 6:**
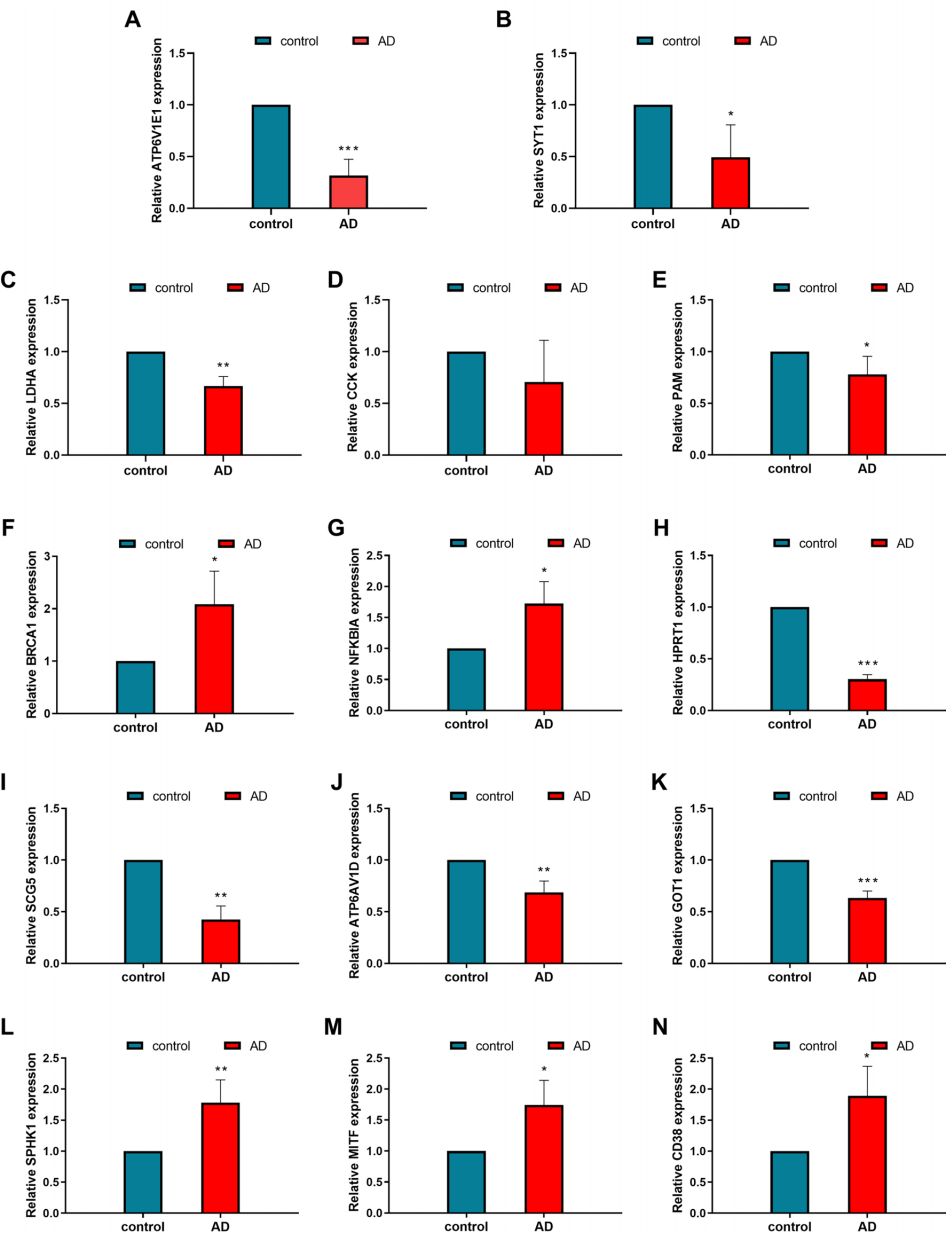
Cerebrospinal fluid PCR expression of 14 genes in AD patients and healthy controls Highly expressed in AD are, CD38(N), MITF(M), SPHK1(L), BRCA1(F), NFKBIA (G). Lowly expressed in AD are, ATP6V1E1 (**A**), SYT1 (**B**), LDHA (**C**), CCK (**D**), PAM (**E**), HPRT1 (**H**), SCG5 (**I**), ATP6AV1D (**J**), GOT1 (**K**).

Consistent patterns were observed in the expression of these genes in the cerebrospinal fluid of both AD patients and healthy controls. Specifically, CCK, ATP6V1E1, SYT1, LDHA, PAM, HPRT1, SCG5, ATP6V1D, and GOT1 exhibited low expression levels in AD patients, whereas NFKBIA, SPHK1, MITF, BRCA1, and CD38 showed high levels of expression in AD patients (Fig. 6).

### Results of in vitro experimental validation

**Figure 7:**
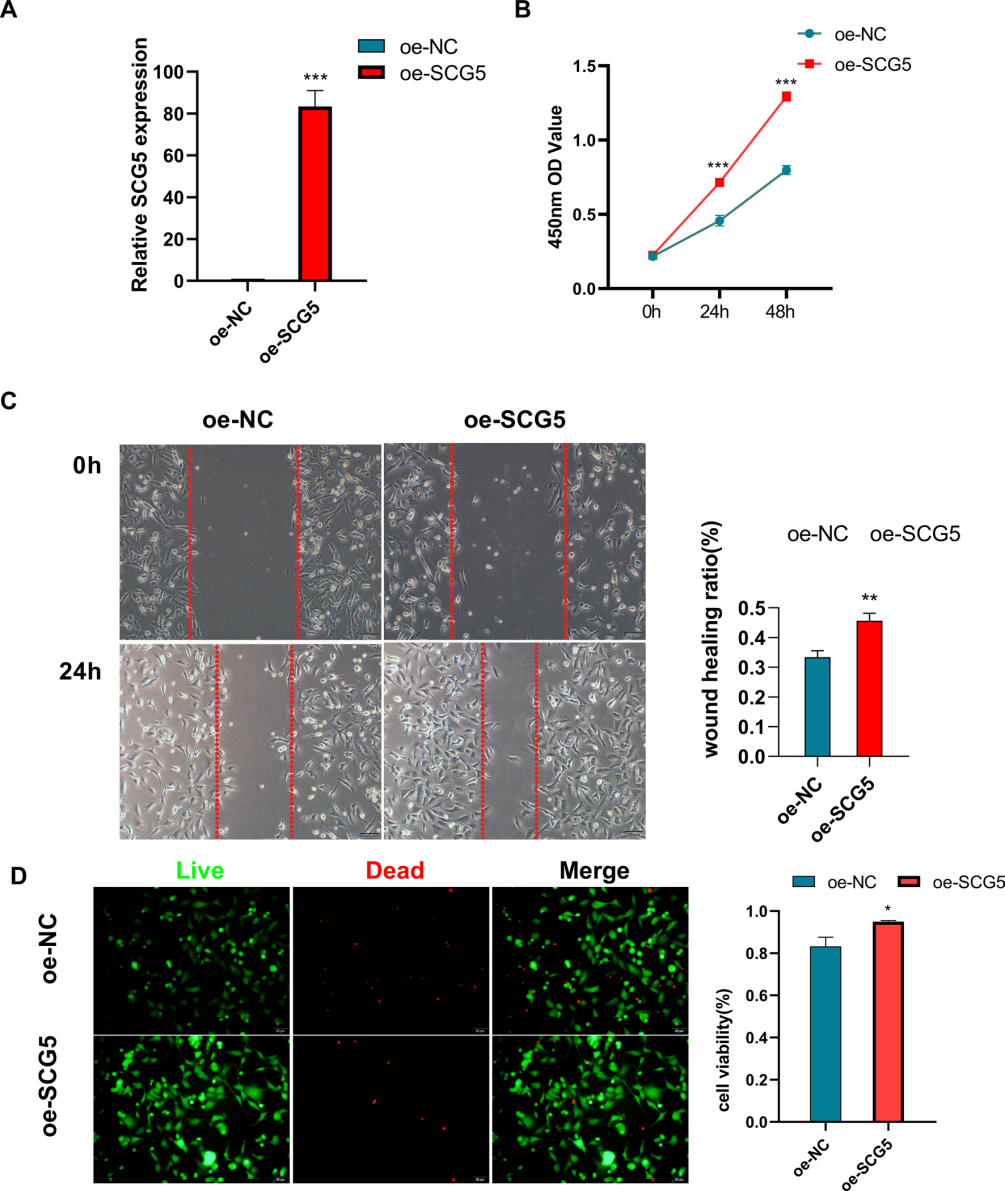
Effects of SCG5 on the HT22 cell line (**A**) SCG5 gene expression was significantly higher in the SCG5 gene over-expression group (oe-SCG5) than in the Over-expression negative control group (oe-NC). (**B**) THE CCK8 assay showed that the OD value at 450 nm was higher in the oe-SCG5 group than in the oe-NC group. (**C**) The migration ability of the oe-SCG5 group was higher than that of the oe-NC group in the Wound Healing experiment. (**D**) Staining of live and dead cells showed that the cell viability of the oe-SCG5 group was higher than that of the oe-NC group.

**Figure 8:**
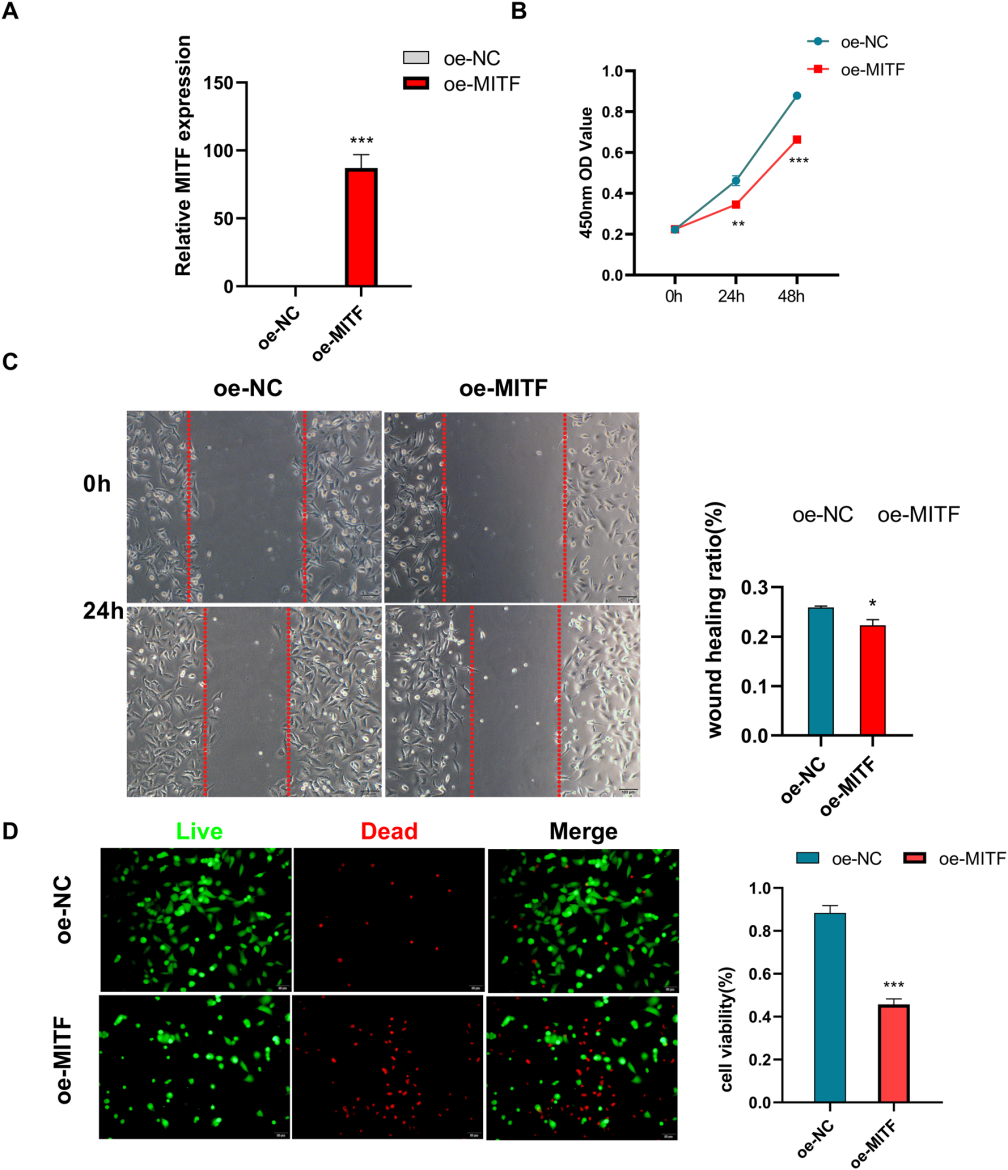
Effect of MITF on the HT22 cell line (**A**) MITF gene expression was significantly higher in the MITF gene overexpression group (oe-MITF) than in the Over-expression negative control group (the oe-NC). (**B**) THE CCK8 assay showed that the OD value at 450 nm in the oe-MITF group was lower than that in the oe-NC group. (**C**) The migration ability of the oe-MITF group was lower than that of the oe-NC group in the Wound Healing experiment. (**D**) Staining of live and dead cells showed that the cell viability of the oe-MITF group was lower than that of the oe-NC group.

Overexpression of SCG5 enhanced the proliferation and migration of HT22 cells, as well as improved the cell viability of HT22 (Fig. 7). In contrast, the overexpression of MITF had a negative impact on the proliferation and migration of HT22 cells, and it also reduced the viability of HT22 cells (Fig. 8).

### The regulatory network for Biomarkers

**Figure 9:**
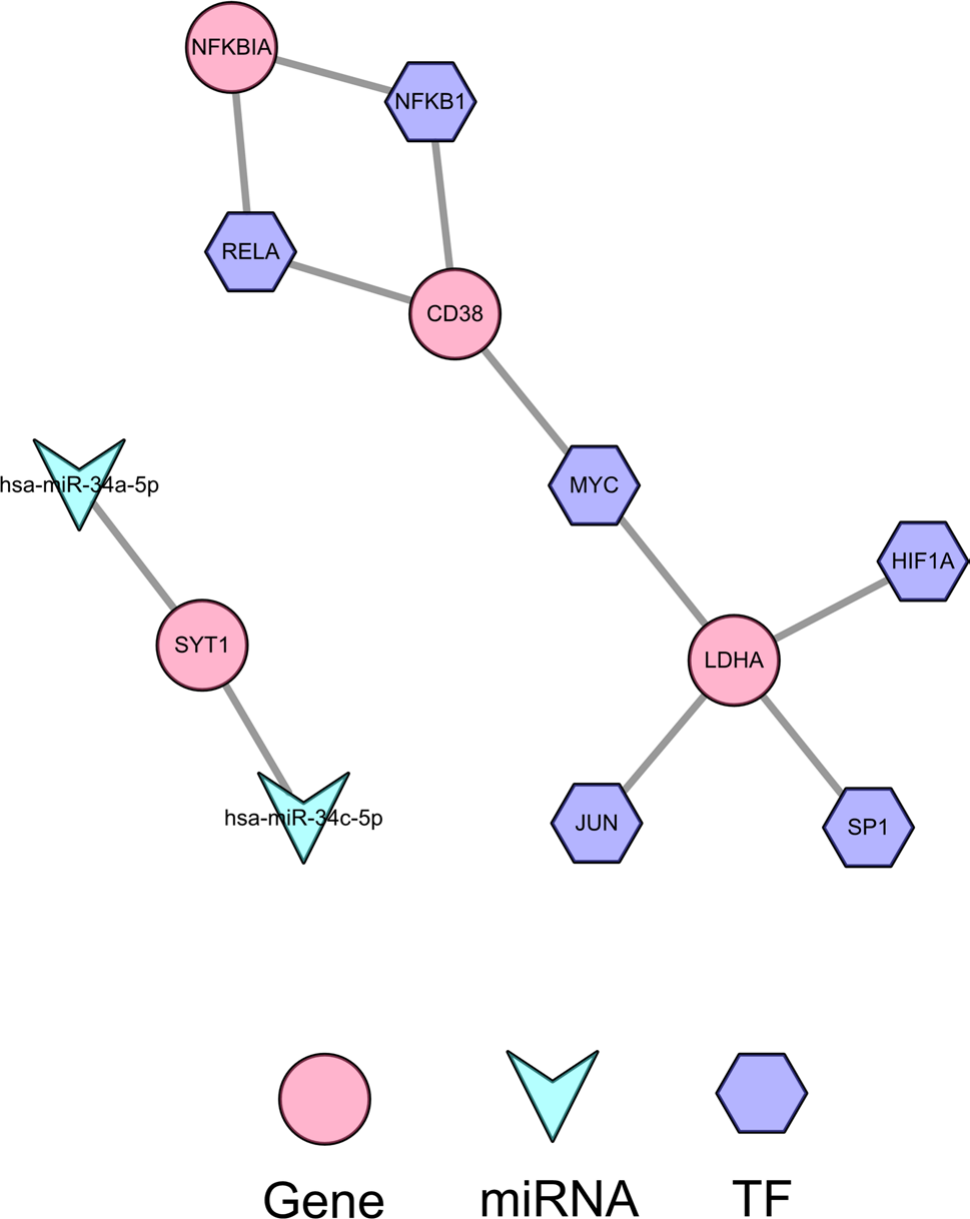
The regulatory network for biomarkers Constructing upstream regulation of final genes based on miRNA-mRNAs and TF-mRNAs.

**Figure 10:**
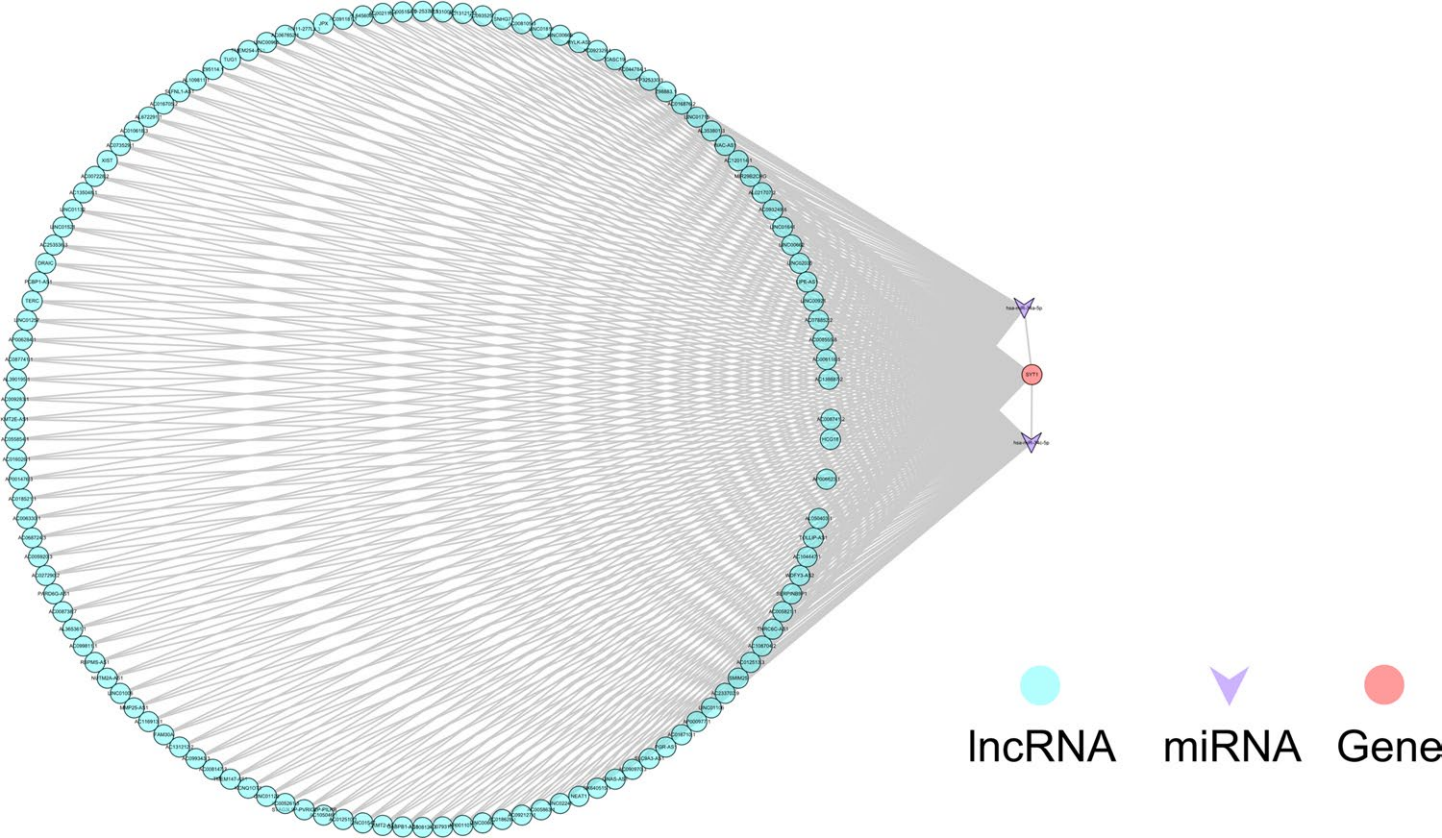
ceRNA regulatory network The lncRNA-miRNA-mRNA co-construction of the ceRNA regulatory network.

Two biomarker-associated miRNAs were mined by prediction from five databases in Starbase, namely hsa-miR-34a-5p and hsa-miR-34c-5p. In addition, six biomarker-associated TFs were predicted through the trust website. Thus, a TF/miRNA-mRNA regulatory network containing 12 nodes (2 miRNAs, 6 TFs, four biomarkers) and 11 edges was established (Fig. 9). In this network, hsa-miR-34a-5p and hsa-miR-34c-5p regulated SYT1. NFKB1 and RELA regulated NFKBIA and CD38. MYC regulated CD38 and LDHA. In addition, HIF1A, JUN, and SP1 modulated LDHA. Then, we predicted lncRNAs targeting hsa-miR-34a-5p and hsa-miR-34c-5p by Starbase. The lncRNAs targeting SYT1 were also expected, and the overlapping of the two predictions was acquired, resulting in 125 lncRNAs. Thus, the lncRNA-miRNA-mRNA network containing 128 nodes (125 lncRNAs, two miRNAs, and one mRNA) and 377 edges was created (Fig. 10, Supplementary Table 7).

### The drug-gene network for biomarkers

**Figure 11:**
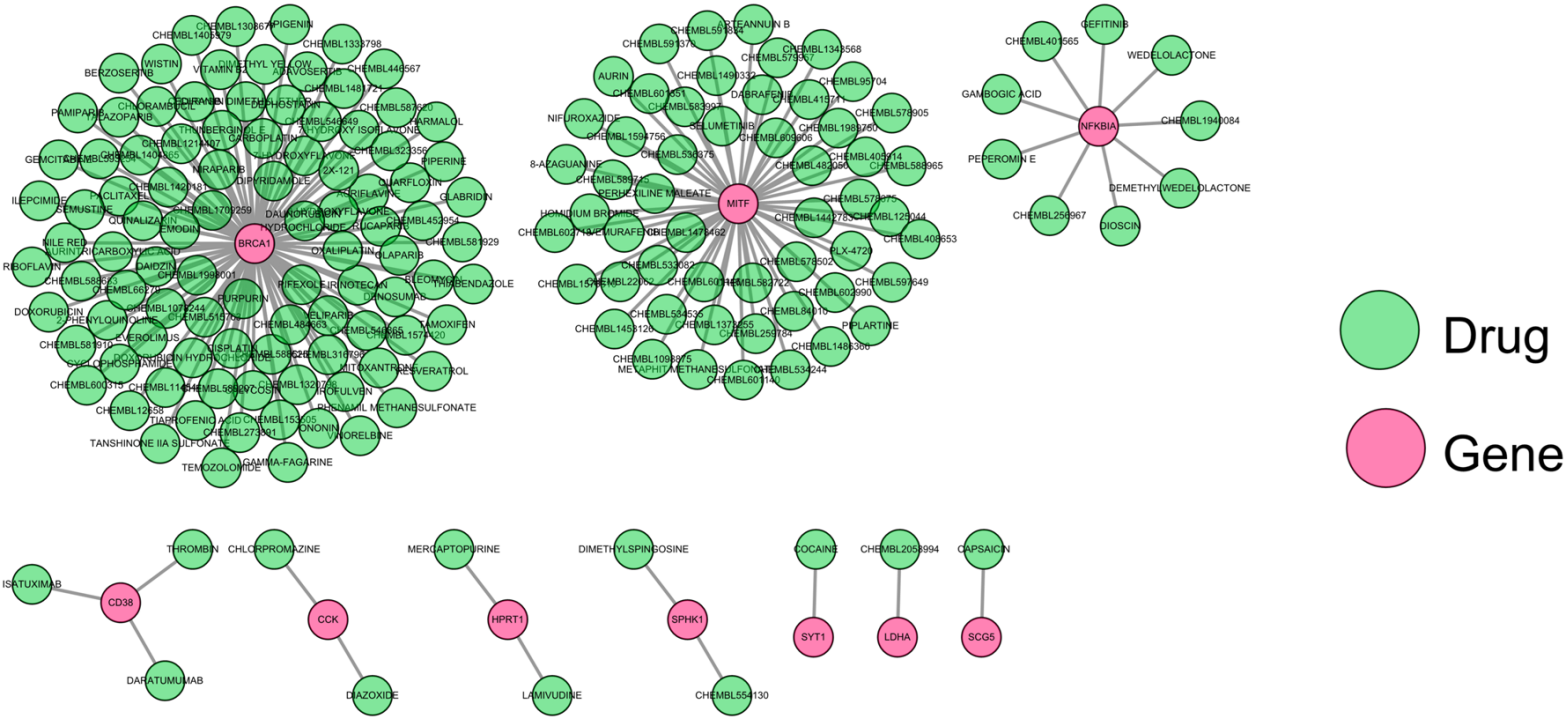
The drug-gene network for biomarkers Through the DGIdb database, 171 medications were aimed at 10 biomarkers.

To initially explore potential drugs targeting the 14 copper metabolism-relevant biomarkers, we predicted 171 drugs targeting ten biomarkers through the DGIdb database (Supplementary Table 8). As exhibited in the drug-gene network, 97 drugs targeted BRCA1, 53 drugs targeted MITF, nine drugs targeted NFKBIA, three drugs targeted CD38, two drugs targeted HPRT1, two drugs targeted SPHK1, and one drug each targeted LDHA, SCG5, and SYT1 (Fig. 11).

## Materials and methods

### Gene and datasets collection

GSE1297 and GSE5281 were used to mine AD datasets from the NCBI-GEO database. The GSE1297 dataset includes microarray expression data from 22 AD hippocampi (HIP) samples and nine healthy controls^14^. The GSE5281 dataset includes microarray expression data from 10 AD patient HIP samples and 13 control samples from healthy people^15^. Supplementary Table 1–2 showed clinical data for two datasets. Using the keyword ‘copper metabolism’, Genecards yielded 2073 copper metabolism-related genes (CMRGs).

### Differential expression analysis

Relying on the threshold value as *p-value* ≤ 0.05 and |log_2_FoldChange (FC)|≥ 0.5, we first determined the DEGs between AD and control samples through ‘limma’ package (version 3.48.3)^16,17^. The DE-CMRGs in AD were acquired by overlapping the DEGs and CMRGs.

### Weighted gene co-expression network analysis (WGCNA)

We filtered AD-associated DE-CMRGs by WGCNA. A co-expression network was created using the R package ‘WGCNA’ (1.70–3)^18^. WGCNA used AD and control samples as trait data to find AD genes. Outliers were removed using sample clustering. To achieve scale-free distribution, a soft-thresholding power was calculated for the co-expression network. The dynamic tree-cutting algorithm was used to create modules with 1 gene each. We set MEDissThres to 0.2 to merge similar modules. The correlation between ultimate modules and traits (AD and control samples) was examined. Critical modules were those with the highest positive and negative AD correlations.

### Functional annotation analysis

The R package ‘clusterProfiler’ (version 4.0.2)^19^ was used for GO and KEGG enrichment analysis^20–22^. The GO was divided into cellular components (CC), molecular functions (MF), and biological processes (BP). The threshold was p < 0.05.

### Immune cell infiltration analysis

The fraction of 24 type immune cells in each AD and control sample from the GSE1297 dataset were calculated based on the ssGSEA algorithm (GSVA package, version 1.40.1)^23^ and 24 immune cell gene set. The correlation between different immune cells was computed by the Pearson method.

### Short time-series expression miner (STEM) clustering

The STEM software was employed for gene clustering based on the expression pattern of each gene at each time point^24^. The gene sets that showed an increasing or decreasing trend over time (*p-value* < 0.001 was defaulted as significant in STEM) were searched for according to the software default parameters, and these gene sets were considered clusters of genes that changed notably with AD development.

### Vivo verification

This study included a total of five patients diagnosed with Alzheimer’s disease and five healthy persons selected from Liaocheng Hospital of Shandong First Medical University. The study (NO.2023039) was authorized by the Ethics Committee of Liaocheng Hospital of Shandong First Medical University. The subjects or their legal guardians provided informed consent. The peripheral blood specimens were subjected to RNA extraction using Trizol reagent (R0016, Beyotime, China). The RNA was converted into complementary DNA (cDNA) using a commercially available reverse transcription kit (11150ES, Yeasen, China) and analyzed using real-time quantitative PCR with a SYBR premixed ELISA. The SYBR premixed Ex Taq kit (AG11718, AG, China) and specific primers were utilized for real-time quantitative PCR detection, as outlined in Table 1. The reference gene employed was glyceraldehyde 3-phosphate dehydrogenase (GAPDH). A statistical analysis was judged significant if the P value was less than 0.05. All methodologies were executed in adherence to the applicable guidelines and regulations., Clinical information on these participants is provided in Table 2**.**

**Table 1:** Primers used for RT-PCR in this study.

Gene	Forward primer (5′-3′)	Reverse primer (5′-3′)
GAPDH	TGCACCACCAACTGCTTAGC	GGCATGGACTGTGGTCATGAG
CCK	AGCTCCTTCTGGACGAATGTC	TGTAGTCCCGGTCACTTATCC
ATP6V1E1	AACATAGAGAAAGGTCGGCTTG	GACTTTGAGTCTCGCTTGATTCA
SYT1	GTGAGCGAGAGTCACCATGAG	CCCACGGTGGCAATGGAAT
LDHA	ATGGCAACTCTAAAGGATCAGC	CCAACCCCAACAACTGTAATCT
PAM	TACCACCAGACCCGTAGTTCC	GTTTAGGTGTAACCCCAGGCA
HPRT1	CCTGGCGTCGTGATTAGTGAT	AGACGTTCAGTCCTGTCCATAA
SCG5	GGGTCCTTTTGGCAACATCC	CCCCTGATCCTCACTAAAGTCC
ATP6V1D	AGCAGGTGTTACTTTGCCAGT	AGTTCCACCAGTAGTTCCACT
GOT1	ATGGCACCTCCGTCAGTCT	AGTCATCCGTGCGATATGCTC
NFKBIA	CTCCGAGACTTTCGAGGAAATAC	GCCATTGTAGTTGGTAGCCTTCA
SPHK1	GCTCTGGTGGTCATGTCTGG	CACAGCAATAGCGTGCAGT
MITF	CAGTCCGAATCGGGGATCG	TGCTCTTCAGCGGTTGACTTT
BRCA1	GAAACCGTGCCAAAAGACTTC	CCAAGGTTAGAGAGTTGGACAC
CD38	AGACTGCCAAAGTGTATGGGA	GCAAGGTACGGTCTGAGTTCC

**Table 2:** General clinical characteristics of the participants.

Samples	gender	age	Sample type
AD	M	65	peripheral blood
AD	M	64	peripheral blood
AD	F	58	peripheral blood
AD	F	59	peripheral blood
AD	M	63	peripheral blood
control	F	59	peripheral blood
control	M	58	peripheral blood
control	M	62	peripheral blood
control	F	63	peripheral blood
control	F	63	peripheral blood

### Cell culture and transfection

The human HT22 cells were acquired from iCell Bioscience, located in Shanghai, China. HT22 cells were cultivated in DMEM media (Gibico, USA) supplemented with 10% FBS (Gibico, USA). The overexpression vectors for SCG5 and MITF, as well as the empty control vectors, were acquired from Shanghai Genechem Co., Ltd. Lentiviral packaging was carried out using the GM easyTM kit from Genomeditech, Shanghai. HT22 cells were infected with lentivirus and then screened using puromycin-containing medium for subsequent cellular function experiments.

### Effect of SCG5/MITF on HT22 cells

The cell proliferation capacity was assessed using the cell counting-8 (CCK8) assay. HT22 cells were divided into groups and seeded into 96-well plates, which were then placed in a cell culture incubator set at 37 degrees Celsius. After 24, 48, and 72 h of incubation, the absorbance of each well was measured by treating the cells with CCK-8 solution for a duration of 2 h. The migratory ability of HT22 cells was assessed using a cell scratch assay, as reported in a previous study^25^. Additionally, the vitality of HT22 cells was evaluated by staining to determine the impact of biomarker. A Live/Dead Cell kit (manufactured by YEASEN, China) was used. The cell viability of the HT22 cell line was evaluated using a Live/Dead Cell kit from YEASEN, China. The fluorescent microscope used to capture the images was from OLYMPUS, Japan.

### Development of TF/miRNA-mRNA and lncRNA-miRNA-mRNA regulatory network

The Starbase was deployed to predict the target miRNAs of critical genes. Then the Starbase was employed to predict lncRNAs targeting the miRNAs and essential genes. The trust website (https://www.grnpedia.org/trrust/) was then utilized to predict the transcription factors (TFs) regulating critical gene expression. The final TF/miRNA-mRNA network and lncRNA-miRNA-mRNA network were constructed using Cytoscape software (version 3.8.2)^26^.

### Development of gene-drug network

The drug-Gene Interaction database (DGIdb) predicted the drugs that targeted the essential genes. Cytoscape (version 3.8.2) was used to generate the final gene-drug network diagram.

### Statistical analysis

The statistical investigation was performed utilizing the R programming language and SSPS.25 software. The Protein–Protein Interaction (PPI) network was built on STRING (https://string-db.org)^27^ with combined_score > 0.4 and enhanced by Cytoscape (version 3.8.2). The pROC package^28^ calculated each gene’s ROC curve AUC. Data from different groups was compared using the Wilcoxon or student’s t-test. Unless otherwise stated, p-values below 0.05 were significant.

### Ethics statement

This study was reviewed and approved by the Institutional Ethics Committee of Liaocheng Hospital, Shandong First Medical University (NO.2023039).

## Discussion

The clinical significance of identifying genes associated with copper metabolism in AD lies in its potential for early intervention in AD. There has been a growing focus on the mechanisms implicated in copper metabolism and homeostasis in the development of AD. In the present investigation, a thorough bioinformatics analysis was performed to ascertain copper metabolism-related biomarkers that are linked to the progression of AD.

The results of functional enrichment analysis revealed that differentially expressed candidate genes associated with AD exhibited a notable pattern of increased expression as the disease progressed. These genes were primarily involved in biological processes related to reactive oxygen species metabolism, immune response, and neuronal apoptosis. The generation of reactive oxygen species (ROS) has the potential to induce impairment in mitochondrial function and diminish the effectiveness of antioxidant defense mechanisms. Consequently, these processes can have an impact on neuronal activity and contribute to cognitive dysfunction, which has been strongly associated with the progression of AD^29^. Neuroinflammation is a characteristic pathological manifestation observed in AD, and the interplay between the central nervous system and the peripheral immune system has been linked to the initiation and progression of AD^30^. The activation of Aβ has been observed to stimulate the intrinsic apoptotic pathway within mitochondria, resulting in the induction of neuronal apoptosis in both brain and neuronal cultures^31^. Thus, it is postulated that the aforementioned genes might potentially play a role in the advancement of diseases via the biological mechanisms elucidated earlier. The downregulation of AD-associated DE-CMRGs, which are primarily linked to neurotransmitter transport-related biological processes, the synaptic vesicle cycle, and GABAergic synapse, is commonly observed during the progression of the disease. The dysregulation of the aforementioned biological processes is intricately linked to the initiation and advancement of AD^32^.

We found 14 copper metabolism-related biomarkers associated with AD progression, including CCK, ATP6V1E1, SYT1, LDHA, PAM, HPRT1, SCG5, ATP6V1D, and GOT1, which decreased with disease severity and may inhibit AD progression. NFKBIA, SPHK1, MITF, BRCA1, and CD38 expression increased with disease severity, which may promote AD progression. CCK (cholecystokinin), a neuropeptide highly expressed in the cerebral cortex, is essential for learning and memory and involved in neurodegeneration^33^. Our findings support previous research that CCK levels protect against AD^34^. CCK is also important for AD hippocampal cognition, according to Liu et al.^35^. SYT1 (synaptotagmin 1) is involved in neurotransmission^36^, and its C2B domain’s binding affinity to lipids is significantly affected by Cu^2+^^37^ and also affects lipid binding^38^. SYT1 is essential for the release of neurotransmitters from hippocampal neurons. Studies conducted on mice have shown that decreases in SYT1 result in impairments in the perception of importance and memory, which worsen the progression of Alzheimer’s disease^36^. Increased expression of LDHA (lactate dehydrogenase A) in lactate production leads to resistance to Aβ and other neurotoxins^39^. In a mouse model of AD, lactate deficiency damages neurons^40^. LDHA may protect against AD progression, as shown by our findings. For amidated neuropeptide synthesis, PAM (peptidylglycine alpha-amidating monooxygenase) is a highly conserved copper-dependent enzyme^41^. Wand et al. found reduced PAM in Alzheimer’s type dementia (DAT) patients’ cerebrospinal fluid^42^. Only the temporal pole showed reduced PAM activity in AD patients compared to controls^42^.

NFKBIA (NFKB inhibitor alpha), linked to immunity and inflammation, is elevated in AD^43,44^ and may contribute to AD development by inhibiting the NF-κB signaling pathway^45^. SPHK1 (sphingosine kinase 1) has been implicated in AD pathogenesis^46^. Takasugi et al. found that SPHK1 inhibitors reduced Aβ production^47^, while Ceccom et al. found that reduced SPHK1 expression in neurons was linked to decreased brain tissue in AD patients^47^. Ceccom et al. found that decreased SPHK1 expression in neurons was linked to Aβ deposition in AD patients’ brain tissue^46,48^. We found that SPHK1 expression was higher in HIP tissues of human AD patients than controls and increased with disease severity. This contradictory result suggests that SPHK1’s role may be cell- and tissue-specific and needs further study.

The BRCA1 (BRCA1 DNA repair associated) gene, which is involved in DNA repair, has been found to be linked to neuronal cell death in AD^49^. Additionally, there is evidence suggesting that BRCA1 may play a role in regulating the re-entry of neuronal cells into the cell cycle in AD^50^. The intricate involvement of CD38 in the processes of neurodegeneration and neuroinflammation has been elucidated in a study by Blacher et al.^51^. Notably, their findings demonstrate that CD38 deficiency in a mouse model leads to a reduction in AD pathology^52^. These results align with our own research, indicating that CD38 serves as a contributing factor in the development and advancement of AD.

In line with prior research^53^, the current study observed a decrease in HPRT1 and GOT1 levels in patients with AD. The potential processes behind this observation are discussed in another study^54^. The absence of HPRT1 leads to changes in mitochondrial energy metabolism in the brain^55^. Inhibiting GOT1 reduces mitochondrial metabolism and induces a catabolic state. The expression of ATP6V1E1 and ATP6V1D is decreased in Alzheimer’s disease, and their function may be linked to abnormal lysosomal activity^56,57^. Currently, the involvement of SCG5 and MITF in AD remains unreported. This study is the first to identify them as potential biomarkers for AD progression. To confirm this, we conducted PCR experiments using clinical samples.

We have developed upstream regulatory networks, including TF/miRNA-mRNA and lncRNA-miRNA-mRNA networks, to explore the relationship between identified biomarkers and disease progression. These networks serve as a foundation for future investigations into the underlying mechanisms of these genes in AD.

CAPSAICIN, a SCG5-targeting compound, was tested in mice and rats to reduce stress-induced AD-like pathology and cognitive impairments^58^. Findings suggest CAPSAICIN may be a promising AD treatment. The authors’ study^59^ found that inhibiting DIOSCIN on NFKBIA reduces neurotoxicity from Aβ1-42 oligomers, reducing neuronal damage and mortality in Alzheimer’s disease patients. DIOSCIN may reduce Alzheimer’s symptoms. A study found that it regulates RAGE/NOX4-mediated oxidative stress and inflammation pathways^60^. Tanshinone IIA sulfonate, targeting BRCA1, improves cognitive function by modulating Aβ transport in transgenic mouse models of AD^61,62^. It also reduces scopolamine-induced cognitive dysfunction by improving the cholinergic system^63^. It has been established that RESVERATROL exhibits a specific affinity towards BRCA1 and possesses antioxidant and anti-inflammatory properties. Furthermore, this compound exhibits neuroprotective properties, potentially reducing Aβ toxicity and aggregation^64^. Song et al. found that targeting CALYCOSIN to BRCA1 may improve cognitive function in transgenic Alzheimer’s mouse models. This improvement comes from protein kinase C activation. In a mouse model, early intrathecal infusion of BRCA1-targeted EVEROLIMUS restores cognitive function and mood in AD mice. BRCA1-targeted APIGENIN has neuroprotective, anti-amyloidogenic, and neurotrophic effects in a mouse model of AD, according to a peer-reviewed study^65^. EMODIN targeting BRCA1 can activate the protein kinase C signaling pathway to reduce AD^66^. A recent study found that EMODIN can delay amyloid-β peptide 1–42 aggregation and enhance cognitive function in transgenic mice with AD^67^. Chen et al.^68^ found that ONONIN, which targets the BRCA1 gene, protected rats with aluminum chloride-induced AD. Thus, the proposed therapeutic agents have promising AD treatment and prevention.

The present study is subject to certain limitations. This study employs a retrospective analysis of data from public sources, supplemented by clinical samples and cellular function investigations to enhance the credibility and substantiation of our analysis. Nevertheless, this investigation encompassed a limited quantity of clinical samples, necessitating comprehensive clinical trials in order to subsequently validate the findings. In conclusion, we have identified for the first time copper metabolism-related biomarkers associated with AD disease progression, providing a basis for further understanding of the molecular mechanisms of the disease, as well as targets for clinical treatment and prevention of the disease.

### Supplementary Information

Supplementary Table 1.

Supplementary Table 2.

Supplementary Table 3.

Supplementary Table 4.

Supplementary Table 5.

Supplementary Table 6.

Supplementary Table 7.

Supplementary Table 8.

## Data Availability

The datasets utilized in this investigation are accessible through online repositories. The repository/repositories’ names and accession number(s) are available at the following link: https://www.ncbi.nlm.nih.gov/, GSE1297 and GSE5281.
